# Lymphome malin non Hodgkinien primitif du larynx: à propos d'un cas

**DOI:** 10.4314/pamj.v9i1.71192

**Published:** 2011-06-10

**Authors:** Abderrahman Elmazghi, Hanan Elkacemi, Issam Lalya, Hanan Zaidi, Amal Harmouch, Lamia Kanouni, Taieb Kebdani, Khalid Hassouni, Noureddine Benjaafar, Brahim Elgueddari

**Affiliations:** 1 Service de Radiothérapie, CHU Hassan II, Fès, Maroc; 2 Service de Radiothérapie, Institut National d'Oncologie, Rabat, Maroc; 3 Service d'Anatomopathologie, CHU Ibn Sina, Rabat, Maroc

**Keywords:** Cancer, larynx, lymphome non-hodgkinien, primitif

## Abstract

Le lymphome malin non hodgkinien (LMNH) primitif laryngé est une entité rare qui représente moins de 1% des cancers du larynx. Nous rapportons un nouveau cas, chez un homme de 24 ans qui avait présenté une dysphonie et une dysphagie d'installations progressives. La laryngoscopie a révélé une lésion bourgeonnante de la bande ventriculaire gauche. L'histologie a objectivé un lymphome T, CD3 positif, qui a bien évolué après traitement par quatre cures de CHOP (cyclophosphamide, doxorubicine, vincristine, et prednisone), suivies d'une radiothérapie sur le larynx et le médiastin supérieur à la dose totale de 40Gy. Malgré sa rareté relative, cette pathologie de diagnostic difficile, nécessite une vigilance particulière et devrait être gérée selon les tendances actuelles du traitement pour les LMNH ganglionnaires.

## Introduction

Les lymphomes malins non hodgkinien (LMNH) de la région tête et cou sont des affections rares: ils représentent 5% de l'ensemble des tumeurs malignes. Sa localisation primitive au niveau du larynx est exceptionnelle, représentant moins de 1% de toutes les tumeurs du larynx. Moins de 100 cas ont été rapportés dans la littérature [1]. Nous rapportons un nouveau cas chez un patient de 24 ans atteint d′un LMNH primitif du larynx. [Agrave] notre connaissance, c′est le premier cas marocain de LMNH primitif du larynx rapporté dans la littérature.

## Patient et observation

Il s'agit d'un jeune homme de 24 ans, ayant dans ses antécédents pathologiques une tuberculose pulmonaire qui a bien évolué sous traitement antibacillaire de six mois. Il présentait une dyspnée d'aggravation progressive, quelques mois avant son admission dans notre service, associée à une dysphagie aux solides, des sueurs nocturnes, un amaigrissement de 10 kg en six mois. L′état général était conservé avec, en particulier, l'absence de fièvre.

**Figure 1:**
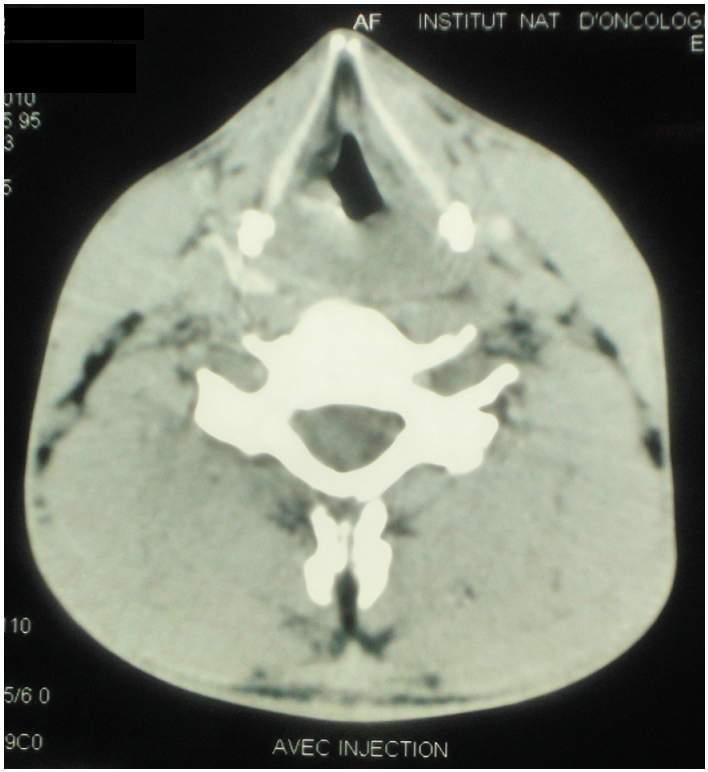
Tomodensitométrie (TDM) du larynx montrant une formation tumorale atteignant les trois étages du larynx avec infiltration partielle de la loge loge hyo-thyro-épiglottique et des deux commissures

**Figure 2:**
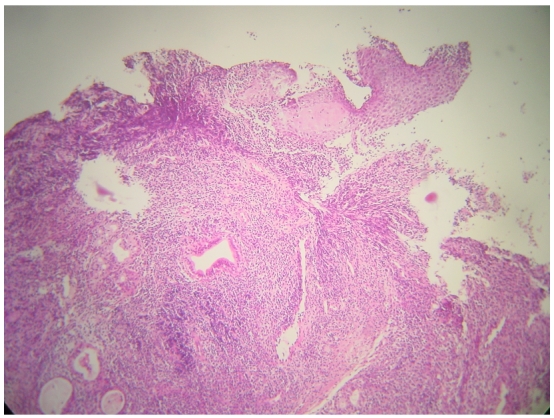
Tématoxyline-éosine (HE) x200: Muqueuse laryngée (épithélium malpighien en haut à gauche) infiltrée par un processus lymphomateux

L'examen clinique était normal et n'a pas trouvé d'adénopathies périphériques. La laryngoscopie mettait en évidence un œdème aryténoïdien bilatéral et une lésion bourgeonnante de la bande ventriculaire gauche dont l’étude histologique après biopsie a objectivé une muqueuse laryngée siège de remaniements inflammatoires, sans signe d'inflammation spécifique ni de malignité. Compte tenu de la normalité du bilan anatomopathologique, une corticothérapie (2 mg /kg/ jour pendant cinq jours) a été instaurée. Elle a fait disparaître la symptomatologie respiratoire. Le patient a ensuite été perdu de vue; la tomodensitométrie(TDM) du larynx prescrite n′a été réalisée que deux mois plus tard. Elle montrait une formation tumorale atteignant les trois étages du larynx avec infiltration partielle de la loge HTE (loge hyo-thyro-épiglottique) et des deux commissures (Figure 1). Une nouvelle laryngoscopie retrouvait un œdème important au niveau des aryténoïdes et des bandes ventriculaires masquant le plan glottique, un aspect grignoté et sale de la bande ventriculaire gauche. La biopsie profonde, trans-muqueuse, a conclu à un lymphome malin non hodgkinien, de type T périphérique (Figure 2).

A l’étude immunohistochimique, les cellules tumorales étaient positives pour le CD3, négatives pour CD20, CD56 et Granzyme. Le bilan biologique était normal (bilan standard dont la numération formule sanguine, les marqueurs de l'inflammation, LDH). Le bilan d'extension (TDM thoraco-abdomino-pelvienne, biopsie osteo-médullaire) était négatif permettant de stadifier la maladie: IEBa de la classification d′Ann Arbor. Le patient a reçu quatre cures de chimiothérapie type CHOP (J1 = J22), avec respect des intervalles inter-cures, suivies d'une radiothérapie, délivrant 40 Gy en 4 semaines, à raison de 5 séances de 2 Gy par semaine, sur le site tumoral et les aires ganglionnaires satellites (y compris le médiastin supérieur), aux photons 6MV d'un accélérateur linéaire, en technique conformationnelle 3D. Les séquelles du traitement ont été minimes. Trois mois après la fin du traitement, la dyspnée et la dysphagie avaient disparu. L′examen endoscopique ainsi que la TDM de contrôle étaient normaux à 6 mois de recul.

## Discussion

Les LMNH du larynx représentent moins de 1% de toutes les tumeurs du larynx. Moins de 100 cas ont été rapportés dans la littérature. L’âge médian de survenue est de 63 ans avec des extrêmes allant de 14 à 90 ans. Les données sur le sex-ratio hommes/femmes sont contradictoires [1–3]. La symptomatologie initiale habituelle comprend une dysphonie, une dysphagie, une sensation de corps étranger, un stridor ou des signes généraux, tels que la perte de poids, les sueurs nocturnes et de la fièvre. Les formes avec détresse respiratoire sont rares. Ces manifestations respiratoires, peuvent faire errer le diagnostic, d'autant plus qu'une corticothérapie précoce et instaurée à l'aveugle peut contribuer à la difficulté diagnostique. Markou a analysé les cas de LMNH primitif de larynx publié entre 1996 et 2008, 47% d′entre eux sont situés dans la région sus-glottique, 25% sont glottiques, le reste sont soit sous-glottique ou transglottique [4]. Sur le plan macroscopique, la plupart des lymphomes du larynx se présentent sous forme d'une masse sous-muqueuse ou d'une tumeur polypoïde, ils sont lisses, non ulcérés, et blanc-grisâtres [3]. Pour affirmer le diagnostic. Il faut souligner que les biopsies doivent fournir un matériel suffisant pour porter le diagnostic histologique. L'immuno-histochimie permet d'affirmer le phénotype B (positivité du CD20, CD19, CD22, des immunoglobulines de surface) ou T (positivité du CD2, CD3) de la cellule maligne. Une analyse cytogénétique ou une étude en biologie moléculaire peut compléter le bilan diagnostique [5]. En se basant sur la classification de l′organisation mondiale de la Santé(OMS) pour les LMNH, Siddiqui a montré que le type B représente 85%, alors que le type T ne représente que 15%. Les LMNH à cellules B sont en outre classés en diffus à grandes, MALT, folliculaire, et le manteau qui ont été observés, respectivement, dans 50%, 20%, 10% et 5% des cas [2].

Le bilan radiologique du LMNH laryngé est celui d'une tumeur des VADS: une TDM cervico-thoraco-abdominale est demandée dans un premier temps, puis l'IRM cervicale qui plus sensible pour l’étude des tissus mous. De façon plus spécifique, une biopsie ostéo-médullaire et un dosage sanguin de LDH sont réalisés. La tomodensitométrie par émission de positons au 18-Fluoro-désoxyglucose (18FDG) couplée au scanner (PET-CT) montre une activité métabolique constante (100%) des cellules lymphomateuses qui est généralement amélioré de façon uniforme avec le contraste iodés (73%) sans nécrose ni calcification [2, 5].

Historiquement, la radiothérapie a été la principale modalité du traitement de ces tumeurs. Elle permet d'obtenir une réponse complète prolongée pour 50 à 90% des patients atteints d'un lymphome stade I ou stade II localisé, mais compte tenu de la nature systémique de la plupart des cas, une chimiothérapie à base de CHOP associe ou non au rituximab selon le type histologique a une place importante, en particulier dans les cas de lymphomes de bas grade [6–9]. Le pronostic des LMNH primitifs du larynx et leur mode d’évolution se rapprochent des LMNH ganglionnaires (atteinte ganglionnaire sus et sous diaphragmatique, envahissement médullaire …). La survie à 10–15 ans est de 50–60% [5].

## Conclusion

Les LMNH de localisation primitive laryngée sont exceptionnels et peuvent évoluer à bas bruit. Cependant la symptomatologie respiratoire peut être au premier plan, et révélatrice de la maladie. Malgré ce caractère exceptionnel, il s'agit d'un diagnostic à évoquer systématiquement. Nous insistons sur l'importance de réaliser des biopsies profondes et d'orienter l'anatomopathologiste vers le diagnostic de LMNH, pour permettre une bonne analyse histologique et immuno-histochimique. Il semble que le LMNH primitif du larynx est une présentation des lymphomes plutôt qu'une maladie à part entière et devrait être géré selon les tendances actuelles de traitement des LMNH ganglionnaires.
